# Body Composition in Acromegaly According to Disease Activity – Performance of Dual X-Ray Absorptiometry and Multifrequency Bioelectrical Impedance Analysis

**DOI:** 10.3389/fendo.2022.866099

**Published:** 2022-05-18

**Authors:** Aline Alves Lopes, Luciano Albuquerque, Mayara Fontes, Daniella Rego, Francisco Bandeira

**Affiliations:** ^1^ Post-Graduate Program in Health Sciences, University of Pernambuco Medical School, Recife, Brazil; ^2^ Clinics Hospital, Federal University of Pernambuco, Recife, Brazil; ^3^ Division of Endocrinology and Diabetes, Agamenon Magalhães Hospital, University of Pernambuco Medical School, Recife, Brazil

**Keywords:** acromegaly, body composition, hormonal control, bioimpedance, DXA (dual x-ray absorptiometry)

## Abstract

**Introduction:**

the present study aims to evaluate body composition and its relationship with hormonal control in acromegaly, also comparing the performance of Bioelectrical impedance analysis (BIA), a more accessible method, with dual X-ray absorptiometry (DXA), technology frequently used in current studies.

**Methods:**

we studied 28 patients (78% female) of whom 13 with active disease, mean age was 52.11 ± 12.53 years; 64% had high blood pressure (HBP) and 50% had type 2 diabetes (T2D).

**Results:**

Although patients with controlled disease had lower serum GH (1.2 ± 1.68µg/L *vs* 6.61 ± 6.40µg/L, p=0.001) and IGF-1 (158.89 ± 54.53ng/mL and 503.31 ± 253.25ng/mL, p<0.001), they did not differ in body composition: percentage of fat mass: 36.13 ± 11.84% *vs* 37.73 ± 8.76%, p=0.691 for BIA and 37.10 ± 10.21% *vs* 37.89 ± 7.32%, p=0.819 for DXA; muscle mass parameters, BIA: FFMI 18.68 ± 2.38kg/m^2^ *vs* 19.14 ± 1.59kg/m^2^, p=0.560; SMI 10.17 ± 1.39kg/m^2^ *vs* 10.53 ± 1.01kg/m^2^, p=0.438; DXA: Baumgartner 7.99 ± 1.43kg/m^2^ *vs* 8.02 ± 1.24kg/m^2^, p=0.947, respectively for controlled and active disease. Patients with controlled acromegaly had lower fasting glucose (110.33 ± 55.48mg/dL *vs* 129.77 ± 40.17mg/dL, p=0.033) and were less likely to have persistent T2D (28.6 vs 71.4%, p=0.008) and HBP (38.9 vs 61.1%, p=0.049). There were strong positive correlations between BIA and DXA for fat mass (r=0.929, p<0.001) and muscle mass parameters: SMI X Baumgartner: r=0.890, p<0.001; and FFMI X Baumgartner: r=0.868, p<0.001.

**Conclusion:**

our data showed similar results in body composition assessment by BIA and DXA, with good correlation between the methods, regardless of the hormonal status of acromegaly. Furthermore, in patients with adequate hormonal control, there was preservation of muscle mass and a lower prevalence of metabolic comorbidities, such as T2D and HBP.

## Introduction

Acromegaly is a rare and slowly progressive disease (1), characterized by excessive secretion of growth hormone (GH) and insulin-like growth factor type 1 (IGF-1) (2), usually caused by a pituitary tumor (1, 2). Due to the increased insulin resistance (3), patients with acromegaly have a high prevalence of metabolic complications such as type 2 diabetes (T2D), high blood pressure (HBP), obstructive sleep apnea syndrome, in addition to an increased risk of other benign and malignant neoplasms (4–9), leading to worsening in the quality of life and high mortality rate (10).

By obtaining hormonal control with normalization of serum IGF-1 for age and GH below 1µg/L (5, 11), the risk of mortality is equal to that of the general population (12). However, the long-term exposure to excess of these hormones causes structural changes, including the modulation of fat deposition and body composition (13–15).

The few data available on body composition in patients with acromegaly have yielded conflicting results (14–18). Some studies have shown that acromegaly patients with active disease have a lower percentage of visceral and subcutaneous adipose tissue (14–18), with increased ectopic distribution of adipose tissue between muscle fibers (16), associated with greater lean mass (14, 15, 18). These findings can be reversed after hormonal control (13–15). However, despite the increase in adipose tissue with normalization of GH/IGF-1, improvements in fasting plasma glucose and hemoglobin A1c have been reported (14, 17).

The present study aims to evaluate body composition in patients with acromegaly, using both dual X-ray absorptiometry (DXA) and multifrequency bioelectrical impedance analysis (BIA), which is portable, cheaper and widely available compared to DXA, and its relationship with hormonal control and metabolic parameters.

## Study Population and Methods

We studied 28 consecutive patients (22 women and 6 men), with a mean age of 52.11 ± 12.53 years and disease duration of 6.43 ± 4.68 years (1 to 20 years). The diagnosis of acromegaly was confirmed by an increase in serum IGF-1 for age, associated with serum GH nadir during an 75g-oral glucose tolerance test (OGTT) above 1µg/L (or above 0.4µg/L for ultrasensitive assays), in the presence of characteristic clinical features (5).

Sample size calculations were not used due to the rarity of acromegaly. Therefore, all patients with acromegaly over 18 years old, either submitted or not to any treatment at Agamenon Magalhães Hospital, who agreed to participate in the study, were included. Patients with uncontrolled endocrine diseases (hyperthyroidism or hypothyroidism), active cancer (except basal cell carcinoma), pregnant women, acute diseases (coronary syndrome or liver failure) as well as chronic renal failure with a glomerular filtration rate below 30 mL/min were excluded.

After signing the informed consent, the patients answered a specific clinical questionnaire and underwent a complete clinical examination; after overnight fasting, blood was collected for laboratory tests and body composition analysis was performed (BIA and DXA), with no database information being used. Serum GH was measured by chemiluminescence using Access UniCel DXI 800 (Beckman Coulter, Rochester, Minnesota, USA), with inter- and intra-assay coefficients of variation < 6%. Serum IGF-1 was measured by chemiluminescence (Immulite 2000, Siemens Healthcare Diagnostics, Germany), with intra- and inter-assay coefficients of variation of 4% and 5.9%, respectively. Serum testosterone and estradiol were also measured by Immulite 2000. Fasting plasma glucose (FPG), glycated hemoglobin (HbA1c), total cholesterol (TC), HDL cholesterol, LDL cholesterol and triglyceride were measured using an auto-analyser (Architect, Abbott, USA).

Body composition analysis was performed by Multifrequency (8-electrode) Bioelectrical impedance (BIA) [Inbody120; Inbody Co., Ltd., South Korea] and total body densitometry (DXA) [Lunar Prodigy; GE Healthcare, Chicago, IL]. The following parameters were used by BIA: fat-free mass index (FFMI: fat-free mass divided by squared height, in kg/m^2^) and skeletal muscle mass index (SMI: total skeletal muscle mass divided by squared height, in kg/m^2^); and by DXA: Baumgartner index (appendicular muscle mass divided by squared height, in kg/m^2^) and Foundation for the National Institutes of Health [FNIH] (appendicular muscle mass divided by BMI). Body fat by both BIA and DXA was expressed as percent fat in relation to body weight. Pituitary imaging was performed by gadolinium-enhanced magnetic resonance (Philips Ingenia 1.5T, Philips Medical Systems LTDA, Netherlands).

The study was carried out in accordance with the Declaration of Helsinki and approved by the Ethics and Research Committee Institution (registration number: CAAE 17520919.0.0000.5197).

### Statistical Analysis

Data were reported using absolute frequencies and percentages for categorical variables and mean and standard deviation for numerical variables. Correlation between two numerical variables was evaluated by Spearman’s correlation coefficient and the corresponding Student’s t-test for the null correlation hypothesis. The comparison between two categories in relation to numerical variables was performed using one of the tests: t-Student with equal variances, t-Student with unequal variances or the Mann-Whitney test. Statistical significance was considered if p<0.05. Data were plotted in Excel spreadsheet and the software used to obtain statistical calculations was the IMB SPSS version 25.

## Results

Of the 28 patients, 23 underwent surgery, with 4 patients cured postoperatively. The remaining 24 patients were using adjuvant drug therapy and 4 underwent radiotherapy (3 remained with active disease). Patients were divided into two groups according to hormonal control (normal IGF-1 for age and GH<1.0 µg/L) (5, 11): active disease versus controlled (inactive) disease. Fifteen patients had hormonal control of GH/IGF-1 (controlled group) and 13 patients had active disease. The general characteristics of the patients are described in **Table 1**. Sixty-four percent of patients had HBP, 14 had T2D, 11 patients had pre-diabetes and 75% had dyslipidemia.

**Table 1:** General characteristics of the study patients (Agamenon Magalhães Hospital, 2021).

Clinical aspect	Inactive (n = 15)	Active (n = 13)	Total (n = 28)	P
**Age (years)**	53.80 ± 12.28	50.15 ± 13.02	52.11 ± 12.53	0.453^(1)^
**Time since diagnosis (years)**	6.2 ± 3.71	6.69 ± 5.75	6.43 ± 4.68	0.787^(1)^
**BMI (kg/m^2^)**	29.61 ± 5.79	31.05 ± 3.41	30.28 ± 4.80	0.438^(1)^
**Abdominal circumference (cm)**	101.10 ± 17.26	98.85 ± 9.81	100.05 ± 14.09	0.681^(1)^
**SBP (mmHg)**	132.00 ± 15.68	132.69 ± 22.04	132.32 ± 18.53	0.786^(2)^
**DBP (mmHg)**	84.67 ± 10.60	83,46 ± 11.44	84.11 ± 10.81	0.775^(1)^
**GH (µg/L)**	1.20 ± 1.68	6.59 ± 6.40	3.70 ± 5.21	0.001^(2)^
**IGF-1 (ng/mL)**	158.89 ± 54.53	503.31 ± 253.25	318.80 ± 246.26	<0.001^(3)^
**FPG (mg/dL)**	110.33 ± 55.48	129.77 ± 40.17	119.36 ± 49.10	0.033^(2)^
**HbA1C (%)**	6.29 ± 1.29	6.73 ± 1.31	6.50 ± 1.30	0.145^(2)^
**Total Cholesterol (mg/dL)**	196.00 ± 34.40	179.85 ± 46.39	188.50 ± 40.47	0.301^(1)^
**HDL-cholesterol (mg/dL)**	49.87 ± 11.32	45.31 ± 10.93	47.75 ± 11.18	0.290^(1)^
**LDL-cholesterol (mg/dL)**	123.93 ± 32.55	104.38 ± 45.40	114.86 ± 39.55	0.198^(1)^
**Triglyceride (mg/dL)**	116.07 ± 47.99	148.77 ± 69.27	131.25 ± 60.02	0.154^(1)^
Current tumor size (cm)*	0.96 ± 0.49	1.92 ± 1.32	1.42 ± 1.07	0.053^(3)^
Initial tumor size (cm)**	1.94 ± 0.81	2.89 ± 1.23	2.38 ± 1.11	0.021^(1)^

Data expressed as mean ± standard deviation. (1): T-student with equal variances; (2): Mann-Whitney test; (3) t-Student with unequal variances. *Current tumor refers to the tumor size at study entry. **Initial tumor refers to the tumor size at the diagnosis. BMI, body mass index; SBP, systolic blood pressure; DBP, diastolic blood pressure; GH, growth hormone; IGF-1, insulin-like growth factor-1; FPG, fasting plasma glucose; HbA1C, glycated hemoglobin.

Regarding the treatment of acromegaly, 24 patients used adjuvant medical therapy: 21 patients were on cabergoline (CAB) plus first-generation somatostatin receptor ligands (SRL) [15 were on Octreotide LAR, with a median dose of 30mg/month, and 6 were on Lanreotide Autogel, with a median dose of 120mg/month]. Three patients used exclusively CAB with a median dose of 2mg/week (0.5 to 3.5mg/week). All patients received at least one type of treatment (surgical, medical or radiotherapy) and none of the patients used pegvisomant or pasireotide. The groups did not differ in terms of diagnosis time: 6.2 ± 3.71 years *vs* 6.69 ± 5.75 years, p = 0.787, respectively for inactive and active groups. Patients with active disease have remained on adjuvant treatment since diagnosis, while the group with controlled disease (inactive) has had adequate hormonal levels for the last 2,8 years (4 patients were cured postoperatively and 11 have undergone adjuvant medical therapy).

**Figure 1:**
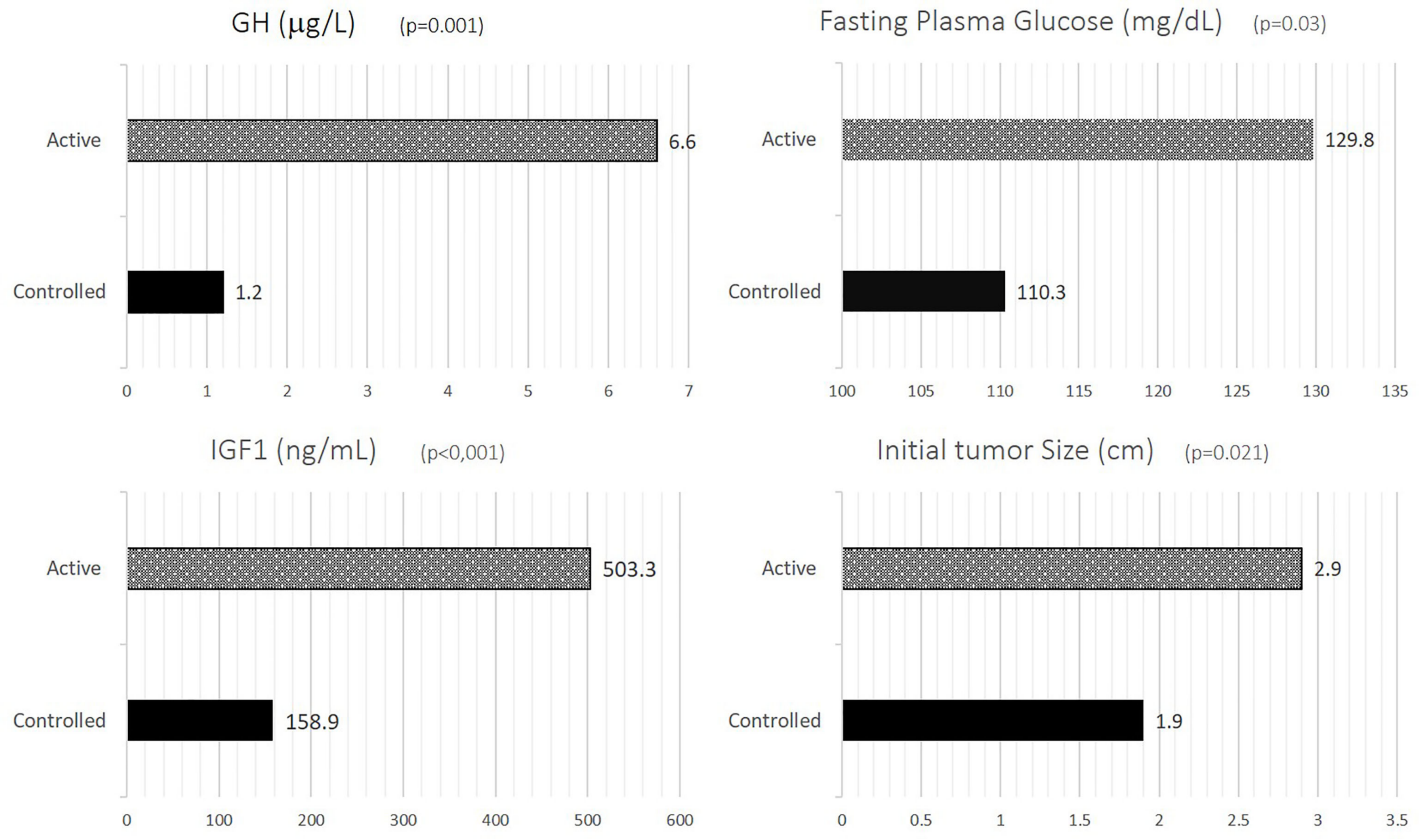
GH, IGF-1, FPG and initial tumor size according to disease activity. GH, growth hormone; IGF-1, insulin-like growth factor-1; FPG, fasting plasma glucose.

Patients with active disease had significantly higher serum GH compared to inactive disease (6.61 ± 6.40µg/L *vs* 1.2 ± 1.68µg/L, p=0.001) and IGF-1 concentrations (503.31 ± 253.25ng/mL *vs* 158.89 ± 54.53ng/mL, p<0.001), larger pituitary tumors both at diagnosis (2.89 ± 1.23cm *vs* 1.94 ± 0.81cm, p=0.021) and at study entry (1.92 ± 1.32cm *vs* 0.96 ± 0.49cm, p=0.053) (**Figure 1**).

When stratifying body composition data according to disease activity, there were no significant differences between inactive and active disease, as follows: fat mass (36.13 ± 11.84% *vs* 37.73 ± 8.76%, p=0.691 for BIA and 37.10 ± 10.21% *vs* 37.89 ± 7.32%, p=0.819 for DXA), FFMI (18.68 ± 2.38 kg/m^2^ *vs* 19.14 ± 1.59 kg/m^2^, p=0.560, SMI (10.17 ± 1.39kg/m^2^ *vs* 10.53 ± 1.01kg/m^2^, p=0.438), Baumgartner (7.99 ± 1.43 kg/m^2^ *vs* 8.02 ± 1.24 kg/m^2^, p=0.947) and FNIH (0.75 ± 0.18 *vs* 0.67 ± 0.15, p=0.108), respectively (**Table 2**).

**Table 2:** Body composition analysis according to disease activity.

Technology	Inactive (n = 15)	Active (n = 13)	P
**BIA**			
Fat mass (%)	36.13 ± 11.84	37.73 ± 8.76	0.691^(1)^
FFMI (kg/m^2^)	18.68 ± 2.38	19.14 ± 1.59	0.560^(1)^
SMI (kg/m^2^)	10.17 ± 1.39	10.53 ± 1.01	0.438^(1)^
**DXA**			
Fat mass (%)	37.10 ± 10.21	37.89 ± 7.32	0.819^(1)^
Baumgartner (kg/m^2^)	7.99 ± 1.43	8.02 ± 1.24	0.947^(1)^
FNIH	0.75 ± 0.18	0.67 ± 0.15	0.108^(2)^

Data expressed as mean ± standard deviation. (1): t-Student with equal variances; (2): Mann-Whitney test. BIA, bioelectrical impedance; DXA, dual X-ray absorptiometry; FFMI, fat-free mass index in Kg/m^2^; SMI, skeletal muscle mass index in Kg/m^2^; Baumgartner, Baumgartner index in Kg/m^2^; FNIH, Foundation for the National Institutes of Health.

Patients with inactive disease had significant lower fasting plasma glucose (**Figure 1**), with no significant differences being observed for other metabolic parameters such as BMI, waist circumference, HbA1c, total cholesterol, HDL-cholesterol, LDL-cholesterol, triglycerides, SBP and DBP (**Table 1**).

**Figure 2:**
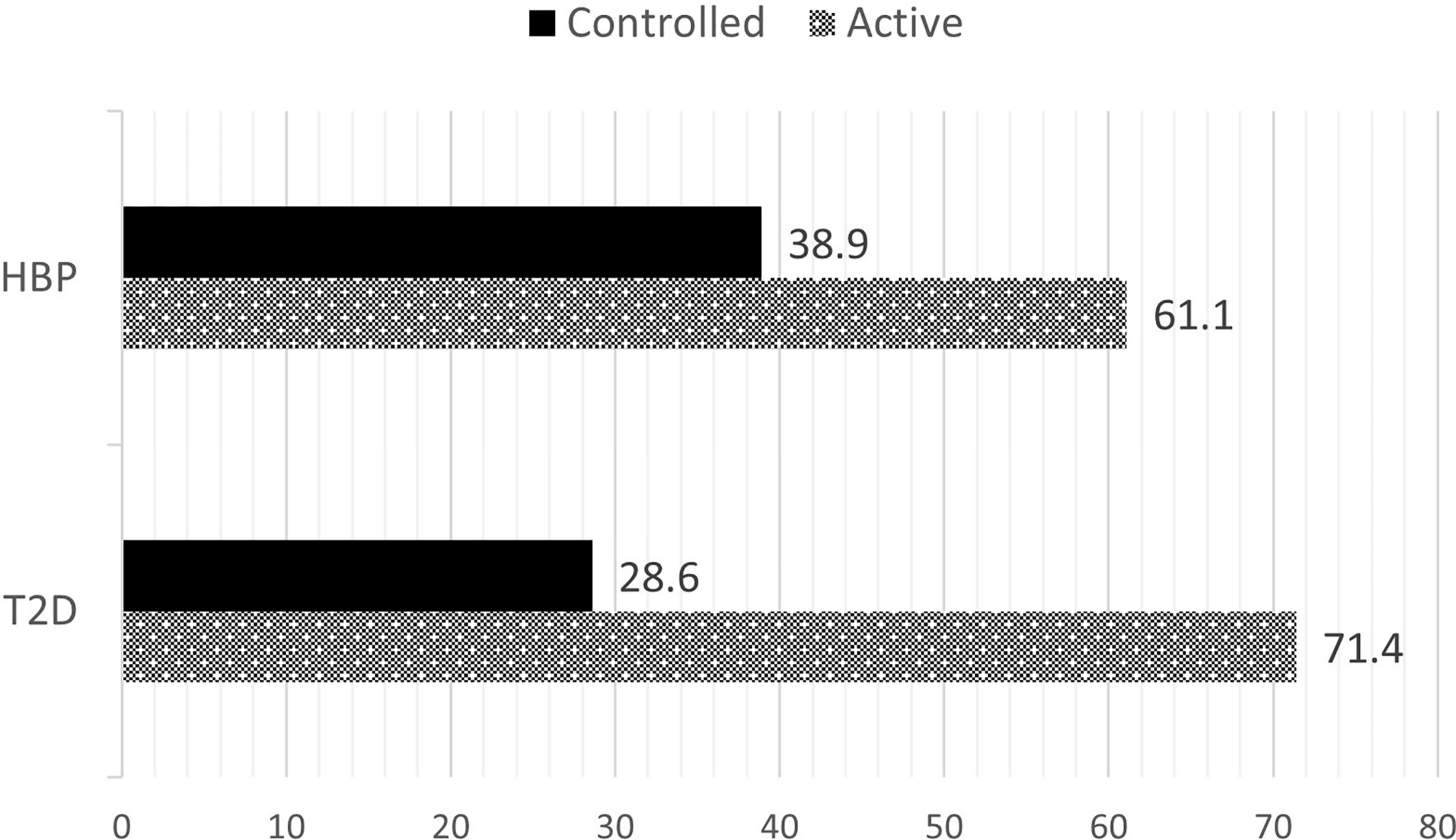
Prevalence of diabetes and hypertension according to disease activity. HBP, high blood pressure; T2D, type 2 diabetes mellitus.

Sixty-one percent of patients with active disease had HBP while 38.9% of patients with inactive disease had persistent hypertension (p=0.049). Similarly, 71.4% of patients with active disease had T2D while 28.6% of patients with inactive disease had persistent T2D (p=0.008) (**Figure 2**).

**Figure 3:**
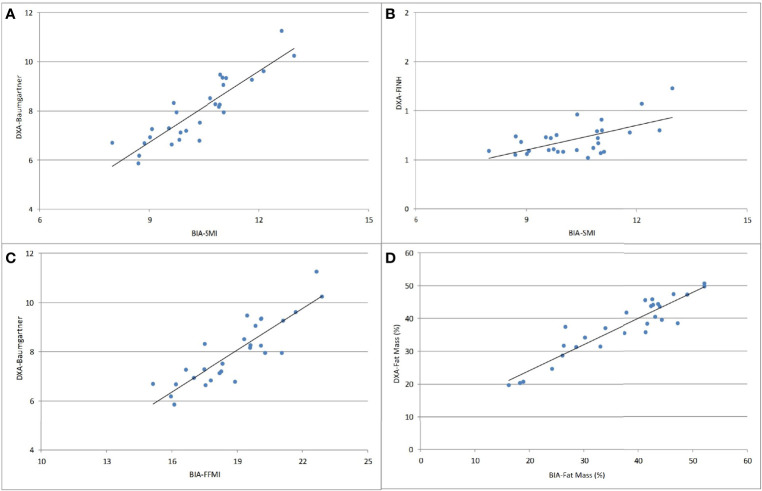
Correlations between BIA and DXA parameters of body composition analysis. **(A)** SMI-BIA x Baumgartner-DXA (r=0.890, p<0.001); **(B)** SMI-BIA x FNIH-DXA (r = 0.498, p = 0.007); **(C)** FFMI-BIA x Baumgartner-DXA (r=0.868, p<0.001); **(D)** fat mass (%) BIA x fat mass (%) DXA (r=0.929, p<0.001). SMI, skeletal muscle mass index; Baumgartner, Baumgartner index; FNIH, Foundation for the National Institutes of Health; FFMI, fat-free mass index.

There were strong positive correlations between BIA and DXA for fat mass (r=0.929, p<0.001) and muscle mass parameters: SMI-BIA X Baumgartner-DXA: r=0.890, p<0.001; and FFMI-BIA X Baumgartner-DXA: r=0.868, p<0.001 (**Figure 3**).

## Discussion

In the present study we demonstrated preservation of muscle mass despite a fall in serum GH/IGF-1 concentrations with disease control in patients with acromegaly. Few studies have addressed changes in body composition in acromegaly and have prioritized the assessment of fat mass, mostly suggesting its increase after obtaining hormonal control (14, 15), differently from the result found in this study. The effects of GH are broad and also extensive to lean tissue, inducing muscle mass gain after replacement in patients who are deficient in this hormone (19); besides due to its lipolytic action and stimulation of protein synthesis, its therapeutic potential in the treatment of obesity has been debated (20).

Different technologies are available for quantification of fat and muscle mass (21–23). Although bioelectrical impedance technology has greatly improved during the last years, few studies have addressed these advances in patients with acromegaly (18), becoming evident that further studies are needed with this medical technology to demonstrate its effectiveness compared to other commonly used methods.

Correlation studies between BIA and DXA have already been performed in other conditions such as primary hyperparathyroidism (22) and following bariatric surgery (23). Similar to their findings, our study showed a strong correlation between DXA and BIA for muscle and fat mass parameters in patients with acromegaly. This may have clinical implications, not only because the structural changes resulting from acromegaly can make the anthropometric measures less accurate, but also because BIA is a portable and less expensive alternative, in addition to being more readily available than other methods such as DXA (15, 23).

Likewise, the literature on studies that assess lean mass in patients with acromegaly is scarce. Lin et al. (14), by using absolute lean mass in kilograms and covering 3 spectrums of disease, demonstrated non-significant long-term changes in patients with acromegaly, including those who developed GH deficiency after treatment: active acromegaly 64.2 ± 3.8kg *vs* acromegaly with GH sufficiency 54.1 ± 2.7kg *vs* acromegaly with GH deficiency 58.5 ± 2.7kg, p = 0.11). These findings may be related to the long period of exposure to excess GH, inducing persistent structural skeletal muscle changes, that led to a non-significant loss of lean mass after hormonal control of acromegaly (14). In the present study we evaluated several indices related to lean and muscle mass by employing two different two different methodologies, with additional assessment by BIA.

Kuker et al. (17) recruited 21 patients with active acromegaly and evaluated their body composition by MRI, before and after pegvisomant. Despite the reduction in lean mass 1 to 2 years after hormonal control, there was no change in skeletal muscle mass. These findings were attributed to the reduction of non-muscle lean tissue which persisted for up to 8 years, even in a situation of “functional GH deficiency” induced by pegvisomant (17).

Likewise, Freda et al. (21) recruited 27 patients with active acromegaly and 315 non-acromegaly control, with the aim of comparing skeletal muscle mass (SM) obtained from total body MRI and the estimated muscle mass (MM) from the DXA, to expand the study of body composition by means of other methods. The authors found a strong correlation between these medical technologies (r = 0.97; p < 0.0001), demonstrating good agreement between them. As a limitation, the authors did not make the same analysis according to hormonal control.

An important point to be highlighted in the present study is the small difference between time of diagnosis and time of hormonal control for inactive patients. This little gap may be responsible for such similar findings in body composition, not having enough time for clinically significant changes. Thus, further studies with long term medical monitoring are needed to assess this hypothesis.

Finally, the present study showed lower rates of hypertension and diabetes in acromegaly patients after hormonal control although still with a high residual risk, as found by others (24, 25). Furthermore, our patients with active disease also had larger pituitary tumors at diagnosis, which is indicative of disease severity, worse surgical outcome and poor long-term prognosis (12, 26, 27).

As a limitation of our study, the cross-sectional design did not allow prospective body composition comparisons before and after hormonal control in individual patient, as well a division by gender, due to a greater number of female patients in this study. As strength, we identified a group of patients with a rare disease, from a single-centre experience, receiving multiple treatment modalities with different degrees of hormonal control, who performed two different technologies for quantification of body fat and muscle, expanding the study of body composition for this group.

## Conclusion

Our data showed similar results in the assessment of body composition in acromegaly by BIA and DXA, regardless of the hormonal status. There was also a strong correlation between the methods, reinforcing the usefulness and reliability of BIA for this purpose. Furthermore, in patients with adequate hormonal control, there was preservation of muscle mass and a lower prevalence of metabolic comorbidities, such as T2D and HBP.

## Data Availability Statement

The original contributions presented in the study are included in the article/supplementary material. Further inquiries can be directed to the corresponding author.

## Ethics Statement

The studies involving human participants were reviewed and approved by Institution Ethics and Research Comittee of Agamenon Magalhães Hospital (registration number: 17520919.0.0000.5197). The patients/participants provided their written informed consent to participate in this study.

## Author Contributions

AAL Data Collection, Data analysis, Manuscript writing and review, Manuscript submission; LA: Data analysis, Manuscript writing; MF: Data Collection, Data analysis; DR: Data Collection, Data analysis, Manuscript writing; FB: Project development, Manuscript writing, editing and review. All authors contributed to the article and approved the submitted version.

## Funding

This study was financed in part by the Coordenação de Aperfeiçoamento de Pessoal de Nível Superior – Brasil (CAPES – Finance Code 001).

## Conflict of Interest

The authors declare that the research was conducted in the absence of any commercial or financial relationships that could be construed as a potential conflict of interest.

## Publisher’s Note

All claims expressed in this article are solely those of the authors and do not necessarily represent those of their affiliated organizations, or those of the publisher, the editors and the reviewers. Any product that may be evaluated in this article, or claim that may be made by its manufacturer, is not guaranteed or endorsed by the publisher.

## References

[1] Dal J Feldt-Rasmussen U Andersen M Kristensen LØ Laurberg P Pedersen L. Acromegaly Incidence, Prevalence, Complications and Long-Term Prognosis: A Nationwide Cohort Study. Eur J Endocrinol (2016) 175(3):181–90. doi: 10.1530/EJE-16-0117 27280374

[2] Colao A Grasso LF Giustina A Melmed S Chanson P Pereira AM. Acromegaly. Nat Rev Dis Primers (2019) 5(1):1–7. doi: 10.1038/s41572-019-0071-6 30899019

[3] Vila G Jørgensen JOL Luger A Stalla GK. Insulin Resistance in Patients With Acromegaly. Front Endocrinol (Lausanne) (2019) 10:509. doi: 10.3389/fendo.2019.00509 31417493 PMC6683662

[4] Petrossians P Daly AF Natchev E Maione L Blijdorp K Sahnoun-Fathallah M. Acromegaly at Diagnosis in 3173 Patients From the Liège Acromegaly Survey (Las) Database. Endocr Relat Cancer (2017) 24(10):505–18. doi: 10.1530/ERC-17-0253 PMC5574208 28733467

[5] Katznelson L Laws ER Jr Melmed S Molitch ME Murad MH Utz A. Endocrine Society. Acromegaly: An Endocrine Society Clinical Practice Guideline. J Clin Endocrinol Metab (2014) 99(11):3933–51 doi: 10.1210/jc.2014-2700. 25356808

[6] Giustina A Barkan A Beckers A Biermasz N Biller BMK Boguszewski C. A Consensus on the Diagnosis and Treatment of Acromegaly Comorbidities: An Update. J Clin Endocrinol Metab (2020) 105(4):dgz096. doi: 10.1210/clinem/dgz096 31606735

[7] Alexopoulou O Bex M Kamenicky P Mvoula AB Chanson P Maiter D. Prevalence and Risk Factors of Impaired Glucose Tolerance and Diabetes Mellitus at Diagnosis of Acromegaly: A Study in 148 Patients. Pituitary (2014) 17:81–9. doi: 10.1007/s11102-013-0471-7 23446424

[8] Gadelha MR Kasuki L Lim DST Fleseriu M. Systemic Complications of Acromegaly and the Impact of the Current Treatment Landscape: An Update. Endocr Rev (2019) 40:268–33. doi: 10.1210/er.2018-00115 30184064

[9] Moller N Jorgensen JO. Effects of Growth Hormone on Glucose, Lipid, and Protein Metabolism in Human Subjects. Endocr Rev (2009) 30(2):152–77. doi: 10.1210/er.2008-0027 19240267

[10] Giustina A Barkhoudarian G Beckers A Ben-Shlomo A Biermasz N Biller B. Multidisciplinary Management of Acromegaly: A Consensus. Rev Endocr Metab Disord (2020) 21(4):667–78. doi: 10.1007/s11154-020-09588-z PMC7942783 32914330

[11] Melmed S Bronstein MD Chanson P Klibanski A Casanueva FF Wass JAH. A Consensus Statement on Acromegaly Therapeutic Outcomes. Nat Rev Endocrinol (2018) 14(9):552–61. doi: 10.1038/s41574-018-0058-5 PMC7136157 30050156

[12] Kasuki L Wildemberg LE Gadelha MR. Management of Endocrine Disease: Personalized Medicine in the Treatment of Acromegaly. Eur J Endocrinol (2018) 178(3):R89–R100. doi: 10.1530/EJE-17-1006 29339530

[13] Katznelson L. Alterations in Body Composition in Acromegaly. Pituitary (2009) 12(2):136–42. doi: 10.1007/s11102-008-0104-8 18369725

[14] Lin E Wexler TL Nachtigall L Tritos N Swearingen B Hemphill L. Effects of Growth Hormone Deficiency on Body Composition and Biomarkers of Cardiovascular Risk After Definitive Therapy for Acromegaly. Clin Endocrinol (Oxf) (2012) 77(3):430–8. doi: 10.1111/j.1365-2265.2012.04361.x PMC3366162 22315983

[15] Reid TJ Jin Z Shen W Reyes-Vidal CM Fernandez JC Bruce JN. IGF-1 Levels Across the Spectrum of Normal to Elevated in Acromegaly: Relationship to Insulin Sensitivity, Markers of Cardiovascular Risk and Body Composition. Pituitary (2015) 18(6):808–19. doi: 10.1007/s11102-015-0657-2 PMC4619193 25907335

[16] Reyes-Vidal CM Mojahed H Shen W Jin Z Arias-Mendoza F Fernandez JC. Adipose Tissue Redistribution and Ectopic Lipid Deposition in Active Acromegaly and Effects of Surgical Treatment. J Clin Endocrinol Metab (2015) 100(8):2946–55. doi: 10.1210/jc.2015-1917 PMC4524994 26037515

[17] Kuker AP Shen W Jin Z Singh S Chen J Bruce JN. Body Composition Changes With Long-term Pegvisomant Therapy of Acromegaly. J Endocr Soc (2021) 5(3):bvab004. doi: 10.1210/jendso/bvab004 33553983 PMC7853172

[18] Guo X Gao L Shi X Li H Wang Q Wang Z. Pre- and Postoperative Body Composition and Metabolic Characteristics in Patients With Acromegaly: A Prospective Study. Int J Endocrinol (2018) 2018:4125013. doi: 10.1155/2018/4125013 29531529 PMC5817290

[19] Lucidi P Laureti S Santoni S Lauteri M Busciantella-Ricci N Angeletti G. Administration of Recombinant Human Growth Hormone on Alternate Days Is Sufficient to Increase Whole Body Protein Synthesis and Lipolysis in Growth Hormone Deficient Adults. Clin Endocrinol (Oxf) (2000) 52(2):173–9. doi: 10.1046/j.1365-2265.2000.00910.x 10671944

[20] Hjelholt A Høgild M Bak AM Arlien-Søborg MC Bæk A Jessen N. Growth Hormone and Obesity. Endocrinol Metab Clinics (2020) 49(2):239–50. doi: 10.1016/j.ecl.2020.02.009 32418587

[21] Freda PU Shen W Reyes-Vidal CM Geer EB Arias-Mendoza F Gallagher D. Skeletal Muscle Mass in Acromegaly Assessed by Magnetic Resonance Imaging and Dual-Photon X-Ray Absorptiometry. J Clin Endocrinol Metab (2009) 94(8):2880–6. doi: 10.1210/jc.2009-0026 PMC2730874 19491226

[22] Voss L Nóbrega M Bandeira L Griz L Rocha-Filho PAS Bandeira F. Impaired Physical Function and Evaluation of Quality of Life in Normocalcemic and Hypercalcemic Primary Hyperparathyroidism. Bone (2020) 141:115583. doi: 10.1016/j.bone.2020.115583 32795678

[23] de Oliveira PAP Montenegro ACP Bezerra LRA da Conceição Chaves de Lemos M Bandeira F. Body Composition, Serum Sclerostin and Physical Function After Bariatric Surgery: Performance of Dual-Energy X-Ray Absorptiometry and Multifrequency Bioelectrical Impedance Analysis. Obes Surg (2020) 30(8):2957–62. doi: 10.1007/s11695-020-04625-x 32335866

[24] Gonzalez B Vargas G de Los Monteros ALE Mendoza V Mercado M. Persistence of Diabetes and Hypertension After Multimodal Treatment of Acromegaly. J Clin Endocrinol Metab (2018) 103:2369–75. doi: 10.1210/jc.2018-00325 29618021

[25] Puglisi S Terzolo M. Hypertension and Acromegaly. Endocrinol Metab Clinics (2019) 48(4):779–93. doi: 10.1016/j.ecl.2019.08.008 31655776

[26] Cuevas-Ramos D Carmichael JD Cooper O Bonert VS Gertych A Mamelak AN. A Structural and Functional Acromegaly Classification. J Clin Endocrinol Metab (2015) 100(1):122–31. doi: 10.1210/jc.2014-2468 PMC4283008 25250634

[27] Bourdelot A Coste J Hazebroucq V Gaillard S Cazabat L Bertagna X. Clinical, Hormonal and Magnetic Resonance Imaging (MRI) Predictors of Transsphenoidal Surgery Outcome in Acromegaly. Eur J Endocrinol (2004) 150(6):763–71. doi: 10.1530/eje.0.1500763 15191345

